# Evaluating the prevalence of current hepatitis C infection and treatment among Aboriginal and Torres Strait Islander peoples who inject drugs in Australia: The ETHOS engage study

**DOI:** 10.1111/dar.13723

**Published:** 2023-08-09

**Authors:** Steven Hobday, Heather Valerio, Troy Combo, Robert Monaghan, Clarke Scott, David Silk, Carolyn Murray, Phillip Read, Charles Henderson, Louisa Degenhardt, Carla Treloar, Gregory J. Dore, Jason Grebely, Marianne Martinello, the ETHOS Engage Study Group

**Affiliations:** ^1^ The Kirby Institute University of New South Wales Sydney Australia; ^2^ Infectious Disease Implementation Science Group Burnet Institute Melbourne Australia; ^3^ Poche Centre for Indigenous Health, University of Queensland Brisbane Australia; ^4^ Nepean Blue Mountains Local Health District, NSW Health Penrith Australia; ^5^ Public Health Programs, NSW Health Sydney Australia; ^6^ Kirketon Road Centre Sydney Australia; ^7^ NSW Users and AIDS Association Sydney Australia; ^8^ National Drug and Alcohol Research Centre, UNSW Sydney Sydney Australia; ^9^ Centre for Social Research in Health University of New South Wales Sydney Australia

**Keywords:** direct acting antivirals, elimination, HCV, Indigenous, people who inject drugs

## Abstract

**Introduction:**

Evaluating progress towards hepatitis C virus (HCV) elimination among Aboriginal and Torres Strait Islander peoples is critical given the disproportionate burden of infection. We examined factors associated with current HCV infection and self‐reported treatment among Aboriginal and Torres Strait Islander (hereafter referred to as Aboriginal peoples) and non‐Aboriginal peoples who inject drugs (PWID) in Australia.

**Methods:**

ETHOS Engage is an observational cohort study of PWID attending drug treatment and needle and syringe programs in Australia. Participants underwent point‐of‐care HCV RNA testing (Xpert HCV RNA Viral Load Fingerstick) and completed a questionnaire including self‐reported history of HCV treatment.

**Results:**

Between May 2018 and June 2021, 2395 participants were enrolled and 555 (23%) identified as Aboriginal (median age 42 years, 58% were men, 63% injected drugs in last month, 76% ever incarcerated). HCV RNA prevalence was 23% among Aboriginal PWID (24% in 2018–2019 and 21% in 2019–2021; *p* = 0.44), and 21% among non‐Aboriginal PWID (24% in 2018–2019 and 16% in 2019–2021; *p* < 0.001). Self‐reported HCV treatment was 65% among Aboriginal PWID (63% in 2018–2019 and 69% in 2019–2021; *p* = 0.30), and 70% among non‐Aboriginal PWID (67% in 2018–2019 and 75% in 2019–2021; *p* < 0.001). Among Aboriginal PWID, current HCV infection was associated with recently injecting drugs and receiving opioid agonist treatment, and self‐reported HCV treatment was negatively associated with younger age, homelessness and recently injecting drugs.

**Discussion and Conclusions:**

Equitable access to HCV care and prevention is needed to ensure Australia meets its elimination targets among Aboriginal PWID.

## 1 INTRODUCTION

Australian Aboriginal and Torres Strait Islander peoples (hereafter respectfully referred to as Aboriginal *) experience significantly poorer health outcomes compared to non‐Aboriginal Australians [1]. Aboriginal peoples face various social, cultural and industrial factors that contribute to inequalities in health, including the lasting impact of colonisation, racism, over‐representation in prisons, limited health literacy, a high proportion of regional and rural residency, and limited access to culturally appropriate health care [2]. In Australia, hepatitis C virus (HCV) infection disproportionately impacts people who inject drugs (PWID), people in prison and Aboriginal peoples [3, 4]. In 2020, 21,584 Aboriginal peoples were estimated to be living with HCV infection in Australia [5], accounting for 18% of those with HCV while comprising 3% of the general population. There has been a sustained upward trend in new HCV notifications among Aboriginal peoples with gaps emerging in access to prevention and treatment [5]. To achieve elimination, populations with high HCV prevalence and incidence will require interventions to optimise testing, linkage to care, treatment and prevention [6].

Direct‐acting antiviral (DAA) therapy has revolutionised the management of HCV infection and given rise to elimination optimism. In 2016, the Australian government listed interferon‐free DAA regimens on the Pharmaceutical Benefits Scheme providing unrestricted government‐subsidised treatment for all adults with HCV infection [7]. The optimism surrounding these therapies catalysed the World Health Organization to call for the elimination of HCV as a global health threat by 2030. Reducing HCV incidence and increasing access to HCV therapy are key to achieving these elimination targets. In Australia, Aboriginal peoples represent priority populations of national and jurisdictional strategies, given increasing rates of infection and liver disease and historically lower treatment uptake in this population [8, 9].

For Aboriginal peoples, HCV infection is predominantly among PWID [10, 11]. Aboriginal peoples are overrepresented among populations at risk of HCV transmission, with compounding harms associated with injecting drug use and incarceration [12, 13, 14]. The intersectionality of racism, discrimination and stigma may limit access to health care for Aboriginal PWID [15, 16, 17]. Understanding the gaps in HCV treatment and disease burden among Aboriginal PWID is essential to achieve parity in health outcomes and equity in the progress towards elimination.

The aims of this analysis were to evaluate the prevalence of and identify the factors associated with current HCV infection and self‐reported history of HCV treatment among Aboriginal PWID attending drug treatment clinics and needle and syringe programs, 5 years following universal access to DAA therapy in Australia.

## 2 METHODS

### 2.1 Study design and participants

The design and methods of ETHOS Engage have been described elsewhere [18, 19]. In brief, ETHOS Engage is an observational cohort study recruiting participants attending needle and syringe programs and drug treatment clinics (including opioid agonist treatment [OAT] clinics and drug and alcohol services) across two recruitment waves: wave 1 (May 2018–September 2019) and wave 2 (November 2019–June 2021). During wave 2, participants were recruited from 21 of the 25 drug treatment clinics (*n* = 19/21) and needle and syringe programs (*n* = 2/4) which participated in wave 1 [18, 19] (Table S1, Supporting Information).

To be included in ETHOS Engage, participants must have been ≥18 years of age, provided written, informed consent and self‐reported injecting drugs, either within the 6 months preceding recruitment or a lifetime injecting history with current OAT. Pregnant participants were excluded from wave 1 of recruitment due to contraindications with transient hepatic elastography (FibroScan®, Echosens, Paris, France); however, the study protocol was amended for wave 2 to include pregnant participants (*n* = 5) given FibroScan was withheld. The initial study protocol and amendments were approved by the Aboriginal Health and Medical Research Council (HREC Ref: 1279/17) and the Human Research Ethics Committees at St Vincent's Hospital, Sydney (HREC Ref: HREC/17/SVH/113).

### 2.2 Procedures

Recruitment consisted of campaign days which occurred between 1 and 5 days per site and included a team of peers, university staff and clinic personnel. After eligibility checks and enrolment, campaign days were run in multiple stages. First, participants underwent point‐of‐care HCV ribonucleic acid (RNA) testing by providing 100 μL finger‐stick capillary whole‐blood to test for HCV RNA using the point‐of‐care Xpert HCV Viral Load Fingerstick Assay (Cepheid, Sunnyvale, United States; lower limit of quantification 100 IU/mL, upper limit of quantification 10^8^ log10 IU/ml; 100% sensitivity, 100% specificity) [20]. Participants then self‐completed a computer tablet‐based questionnaire collecting data on demographics, behavioural risk and HCV treatment experience. Peers were available for assistance with the questionnaire if needed. Liver fibrosis was assessed using FibroScan, after which participants underwent a brief consultation with appropriate clinical staff. Participation was remunerated (30 AUD voucher).

### 2.3 Identifying participants who identified as Aboriginal and Torres Strait Islander peoples

During study enrolment, participants were asked, ‘Do you identify as: (1) Aboriginal, (2) Torres Strait Islander, (3) Both, (4) Neither, (5) Don't know’. Participants who selected (1) (2) or (3) comprised the population who identified as Aboriginal peoples, whereas those who selected (4) and (5) comprised those who did not identify as Aboriginal and/or Torres Strait Islander peoples (non‐Aboriginal).

### 2.4 Outcomes at baseline

The primary outcome was current HCV infection (HCV RNA detected with the Xpert HCV Viral Load Fingerstick assay). The secondary outcome was self‐reported history of HCV treatment (collected from participant survey) among participants who have ever been eligible for HCV therapy, including those with evidence of past (self‐reported history of HCV treatment) or current HCV infection [18, 19]. Participants who were never infected (HCV RNA undetectable and self‐reported as never diagnosed with HCV) and who had spontaneously cleared (HCV RNA undetectable, self‐reported historical HCV diagnosis and self‐reported never receiving HCV treatment) were also identified (Figure S1) [18]. Outcomes were assessed among Aboriginal and non‐Aboriginal PWID separately.

### 2.5 Statistical analysis

Individuals who were enrolled within and between recruitment wave 1 and wave 2 were identified by two‐by‐two name code (first two letters of first name and last name) and date of birth. Among those who were identified as having participated more than once within recruitment waves, the first enrolment was kept. Furthermore, the first enrolment was used for those who were identified as having participated in both recruitment wave 1 and wave 2.

For both outcomes, recruitment wave was the factor of interest (wave 1 [May 2018—September 2019], wave 2 [November 2019—June 2021]). In addition, the demographic and behavioural factors hypothesised to be associated with current HCV infection and HCV treatment were determined using published ETHOS Engage results [18, 19] and previous HCV infection and treatment research focused on Aboriginal peoples [12, 13, 21, 22, 23]. These factors included: (i) age at survey (stratified around total participant median); (ii) gender (man, woman, other [non‐binary/transgender/other]); (iii) location of recruitment site (major cities of Australia, regional Australia); (iv) accommodation in the past 6 months indicating homelessness (no, yes); (v) OAT status (never, past, within the last month/current); (vi) excessive alcohol consumption (defined using AUDIT‐C for men [≥4] and women [≥3] participants) (no, yes) [24]; (vii) incarceration history (never, history only [not recent], recent; as defined by last 12 months in wave 1 and last 6 months in wave 2); (viii) recency and frequency of injecting drug use (>1 year ago, within the previous 1–12 months, within the previous month <daily, and ≥daily); and (ix) main drug injected in the last month (none, heroin, other opioids, methamphetamine, other).

Logistic regression was used to assess the factors associated with: (i) current HCV infection; and (ii) history of HCV treatment among Aboriginal and Torres Strait Islander and non‐Aboriginal participants. For Aboriginal and non‐Aboriginal participants, the association between demographic and behavioural factors and outcomes were analysed in unadjusted analyses. Variables with *p* ≤ 0.30 at the unadjusted level or with known or suspected clinical significance were considered for the adjusted models.

A supplementary analysis was performed for Aboriginal peoples who reported recent injecting drug use (in the month previous to the survey). For these supplementary analyses, recency and frequency of injecting was recategorised (<daily, ≥daily) and main drug injected in the last month was recategorised (heroin, other opioids, methamphetamine, other).

Data cleaning, storage and analyses were conducted using Stata 14.2 (StataCorp, College Station, TX, USA).

## 3 RESULTS

### 3.1 Sample characteristics

The sample characteristics of the overall ETHOS Engage population by Aboriginal identification are presented in Table 1. Overall, 2395 unique participants were enrolled between May 2018 and June 2021: 555 (23%) participants who identified as Aboriginal and/or Torres Strait Islander (Aboriginal, *n* = 512; Torres Strait Islander, *n* = 20; Aboriginal and Torres Strait Islander, *n* = 23) and 1840 (77%) who did not (Table 1, Figure S2). A breakdown of participant characteristics by recruitment wave and Aboriginal identification are presented in Tables S2 and S3.

**TABLE 1:** Sample characteristics of ETHOS Engage participants, by Aboriginal or Torres Strait Islander identification.

		Overall population	Aboriginal and Torres Strait Islander peoples	Non‐Aboriginal
Total		2395	555	1840
Median age (IQR)		43 (37, 50)	42 (36, 49)	44 (37, 51)
Gender	Man	1591 (66%)	324 (58%)	1267 (69%)
	Woman	786 (33%)	229 (41%)	557 (30%)
	Transgender/other^a^	18 (1%)	2 (<1%)	16 (1%)
Location of clinic	Major cities of Australia	1816 (76%)	402 (72%)	1414 (77%)
	Regional Australia	579 (24%)	153 (28%)	426 (23%)
Homeless	No	2134 (89%)	485 (87%)	1649 (90%)
	Yes	261 (11%)	70 (13%)	191 (10%)
OAT status	Never	371 (15%)	100 (18%)	271 (15%)
	Past	305 (13%)	66 (12%)	239 (13%)
	Current	1719 (72%)	389 (71%)	1330 (72%)
Incarceration history	Never	771 (32%)	120 (22%)	651 (35%)
	History only	1181 (49%)	300 (54%)	881 (48%)
	Recent	443 (19%)	135 (24%)	308 (17%)
Excessive alcohol consumption^b^	No	1456 (61%)	339 (61%)	1117 (61%)
	Yes	921 (39%)	214 (39%)	707 (39%)
Recency of injecting	>12 months	334 (14%)	76 (14%)	258 (14%)
	Within 1–12 months	506 (21%)	130 (23%)	376 (20%)
	Within last month, <daily	822 (34%)	177 (32%)	645 (35%)
	Within last month, ≥daily	733 (31%)	172 (31%)	561 (30%)
Main drug injected in last month	None	840 (35%)	206 (37%)	634 (34%)
	Heroin	535 (22%)	108 (19%)	427 (23%)
	Other opioids	201 (8%)	29 (5%)	172 (9%)
	Methamphetamine	780 (33%)	200 (36%)	580 (32%)
	Other	39 (2%)	12 (2%)	27 (1%)
Recruitment wave	Wave 1 (2018–2019)	1443 (60%)	337 (61%)	1106 (60%)
	Wave 2 (2019–2021)	952 (40%)	218 (39%)	734 (40%)

Abbreviations: IQR, interquartile range; OAT, opioid agonist treatment.

^a^ Other refers to any individual who did not identify as man, woman or transgender.

^b^ Not reported for transgender/other.

Among Aboriginal participants (*n* = 555), 58% (*n* = 324) were men, median age was 42 (interquartile range 36, 49), 71% (*n* = 389) were currently receiving OAT and 78% (*n* = 435) had ever been incarcerated; of those who injected drugs in the last month (63%, *n* = 349), most mainly injected methamphetamine (57%, 200/349) (Table 1). Among non‐Aboriginal participants (*n* = 1840) 69% (*n* = 1267) were men, median age was 44 (interquartile range 37, 51), 72% (*n* = 1330) were currently receiving OAT and 65% (*n* = 911) had ever been incarcerated; of those who injected drugs in the last month (66%, *n* = 1206), most mainly injected methamphetamine (48%, 580/1206) (Table 1).

### 3.2 Current HCV infection

**FIGURE 1:**
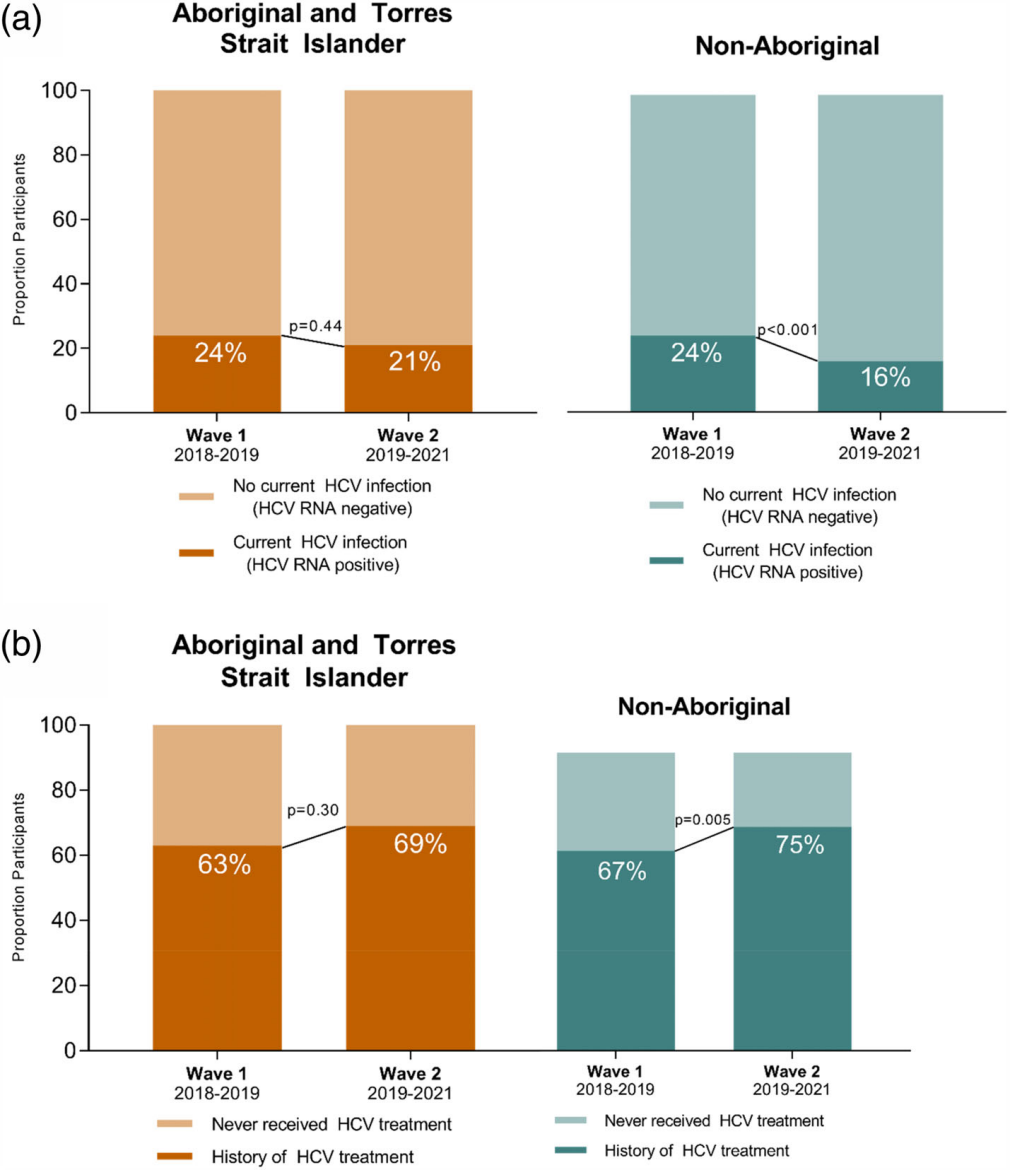
Change in current hepatitis C virus (HCV) infection (a) and self‐reported HCV treatment (b) between ETHOS Engage recruitment waves, by Aboriginal and Torres Strait Islander status. RNA, ribonucleic acid.

Among Aboriginal participants with valid HCV RNA results (*n* = 536), prevalence of current HCV infection (HCV RNA detectable) was 23% (*n* = 123) and did not decline significantly between 2018–2019 and 2019–2021 (24% [78/324] to 21% [45/212]; *p* = 0.44) (Table 2, Figure 1a). Among non‐Aboriginal participants with valid HCV RNA results (*n* = 1769), prevalence of current HCV infection was 21% (*n* = 364) and declined significantly between 2018–2019 and 2019–2021 (24% [253/1064] to 16% [111/705]; *p* < 0.001) (Figure 1a).

**TABLE 2:** Factors associated with current HCV infection among Aboriginal and Torres Strait Islander participants.^a^

Characteristic		Total known HCV RNA result, *n* (col%)	Current HCV RNA infection, *n* (row%)	OR (95% CI)	aOR (95% CI)
Total		536	123 (23%)		
Age at enrolment	<45	325 (61%)	83 (26%)	‐ref‐	‐ref‐
	≥45	211 (39%)	40 (19%)	0.68 (0.45, 1.04)	0.70 (0.45, 1.09)
Gender	Man	309 (58%)	76 (25%)	‐ref‐	‐ref‐
	Woman	225 (42%)	47 (21%)	0.81 (0.54, 1.22)	0.78 (0.50, 1.22)
	Transgender/other^b^	2 (<1%)	0 (0%)	Omitted	Omitted
Location of clinic	Major cities of Australia	338 (63%)	90 (23%)	‐ref‐	
	Regional Australia	148 (37%)	33 (22%)	0.95 (0.60, 1.49)	
Homeless	No	470 (88%)	102 (22%)	‐ref‐	‐ref‐
	Yes	66 (12%)	21 (32%)	1.68 (0.96, 2.95)	1.74 (0.95, 3.18)
OAT status	Never	96 (18%)	14 (15%)	‐ref‐	‐ref‐
	Past	66 (12%)	18 (27%)	2.19 (1.00, 4.81)	2.09 (0.93, 4.68)
	Current	374 (70%)	91 (24%)	1.89 (1.02, 3.48)	2.18 (1.15, 4.14)
Incarceration history	Never	117 (22%)	20 (17%)	‐ref‐	‐ref‐
	History only	291 (54%)	69 (24%)	1.51 (0.87, 2.62)	1.42 (0.79, 2.55)
	Recent	128 (24%)	34 (27%)	1.75 (0.94, 3.26)	1.34 (0.68, 2.62)
Excessive alcohol consumption^c^	No	326 (61%)	69 (21%)	‐ref‐	
	Yes	208 (39%)	54 (26%)	1.30 (0.87, 1.96)	
Recency of injecting	>12 months	74 (14%)	10 (14%)	‐ref‐	‐ref‐
	Within 1–12 months	125 (23%)	25 (20%)	1.60 (0.72, 3.56)	1.68 (0.74, 3.82)
	Within last month, <daily	174 (33%)	43 (25%)	2.10 (0.99, 4.45)	2.37 (1.09, 5.13)
	Within last month, ≥daily	163 (30%)	45 (28%)	2.44 (1.15, 5.16)	2.33 (1.05, 5.17)
Main drug injected in last month	None	199 (37%)	35 (18%)	‐ref‐	
	Heroin	103 (19%)	33 (32%)	2.21 (1.27, 3.83)	
	Other opioids	28 (5%)	6 (21%)	1.28 (0.48, 3.83)	
	Methamphetamine	195 (36%)	47 (24%)	1.49 (0.91, 2.43)	
	Other	11 (2%)	2 (18%)	1.04 (0.21, 5.03)	
Recruitment wave	Wave 1 (2018–2019)	324 (30%)	78 (24%)	‐ref‐	‐ref‐
	Wave 2 (2019–2021)	212 (37%)	45 (21%)	0.85 (0.56, 1.29)	0.83 (0.54, 1.27)

Abbreviations: aOR, adjusted odds ratio; CI, confidence interval; HCV, hepatitis C virus; OAT, opioid agonist treatment; OR, odds ratio; RNA, ribonucleic acid.

^a^ With valid HCV RNA point‐of‐care test results, *N* = 536.

^b^ Other refers to any individual who did not identify as a man, woman or as transgender.

^c^ Not reported transgender/other.

### 3.3 Factors associated with current HCV infection among Aboriginal and Torres Strait Islander participants

**FIGURE 2:**
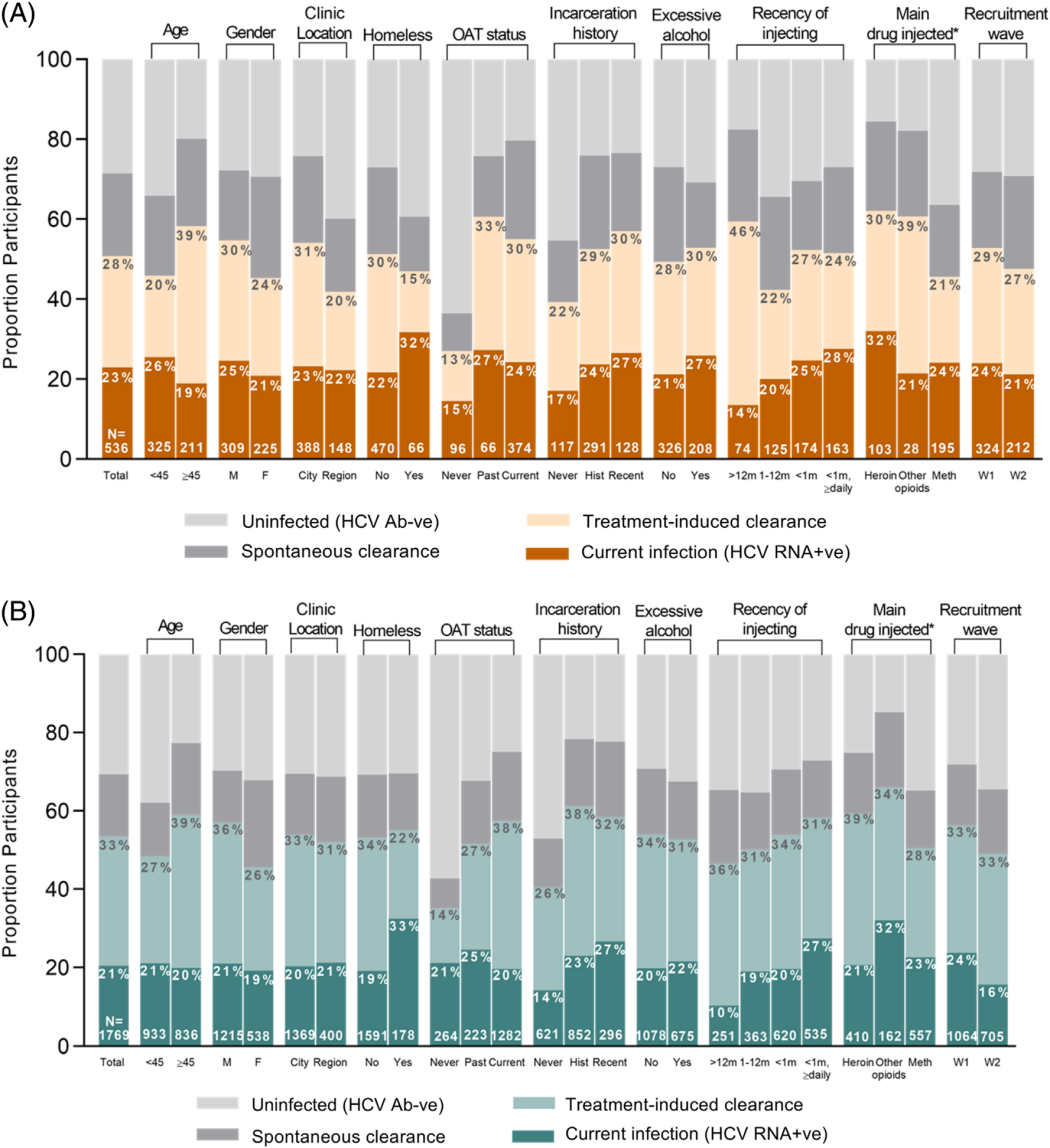
Current HCV infection status among ETHOS Engage participants with valid point‐of‐care hepatitis C virus (HCV) ribonucleic acid (RNA), among Aboriginal and Torres Strait Islander (a) and non‐Aboriginal (b) participants. *Main drug injected in the last month; “other” not shown due to small numbers.

Current HCV infection by characteristics of interest among Aboriginal participants is presented in Table 2 and Figure 2a. In adjusted analyses, recruitment wave was not associated with current HCV infection. In adjusted analyses, current HCV infection was more likely among those who were currently receiving OAT (vs. never received OAT, adjusted odds ratio [aOR] 2.18 95% confidence interval [CI] 1.15, 4.14) and among those who had injected in the previous month (vs. injecting more than 1 year ago; <daily in the previous month aOR 2.37, 95% CI 1.09, 5.13; ≥daily in the previous month aOR 2.33, 95% CI 1.05, 5.17) (Table 2). Among Aboriginal participants who injected drugs in the previous month, frequency and type of drug injected were not associated with current HCV infection (Table S4).

### 3.4 Factors associated with current HCV infection among non‐Aboriginal participants

Current HCV infection by characteristics of interest among non‐Aboriginal participants is presented in Table 3 and Figure 2b. In adjusted analyses, participants recruited between 2019–2021 (wave 2) were less likely to have current HCV infection (vs. 2018–2019 [wave 1] aOR 0.55, 95% CI 0.43, 0.72) (Table 3). In adjusted analyses, current HCV infection was more likely among those who were homeless (aOR 1.75, 95% CI 1.23, 2.50), those who had ever been incarcerated (vs. never incarcerated, history aOR 1.81, 95% CI 1.35, 2.42; recent incarceration aOR 2.14, 95% CI 1.48, 3.08) and those who had injected drugs in the previous 12 months (vs. injecting more than 12 months ago, within previous 1–12 months, aOR 1.94, 95% CI 1.18, 3.18; <daily in the previous month, aOR 1.91, 95% CI 1.20, 3.04; ≥daily in the previous month, aOR 3.09, 95% CI 1.93, 4.91) (Table 3).

**TABLE 3:** Factors associated with current HCV infection among non‐Aboriginal participants.^a^

Characteristic		Total known HCV RNA result, *n* (col%)	Current HCV RNA infection, *n* (row%)	OR (95% CI)	aOR (95% CI)
Total		1769	364 (21%)		
Age at enrolment	<45	933 (53%)	197 (21%)	‐ref‐	‐ref‐
	≥45	836 (47%)	167 (20%)	0.93 (0.74, 1.17)	1.00 (0.78, 1.27)
Gender	Man	1215 (69%)	256 (21%)	‐ref‐	‐ref‐
	Woman	538 (30%)	104 (19%)	0.89 (0.70, 1.16)	1.00 (0.77, 1.31)
	Transgender/other^b^	16 (1%)	4 (25%)	1.25 (0.40, 3.90)	Omitted
Location of clinic	Major cities of Australia	1369 (77%)	279 (20%)	‐ref‐	
	Regional Australia	400 (23%)	85 (21%)	1.05 (0.80, 1.39)	
Homeless	No	1591 (90%)	306 (19%)	‐ref‐	‐ref‐
	Yes	178 (10%)	58 (33%)	2.03 (1.44, 2.84)	1.75 (1.23, 2.50)
OAT status	Never	264 (15%)	56 (21%)	‐ref‐	‐ref‐
	Past	223 (13%)	55 (25%)	1.21 (0.80, 1.86)	1.10 (0.71, 1.72)
	Current	1282 (73%)	253 (20%)	0.91 (0.66, 1.26)	0.98 (0.69, 1.39)
Incarceration history	Never	621 (35%)	89 (14%)	‐ref‐	‐ref‐
	History only	852 (48%)	196 (23%)	1.79 (1.36, 2.35)	1.81 (1.35, 2.42)
	Recent	296 (17%)	79 (27%)	2.18 (1.55, 3.06)	2.14 (1.48, 3.08)
Excessive alcohol consumption^c^	No	1078 (61%)	214 (20%)	‐ref‐	
	Yes	675 (38%)	146 (22%)	1.11 (0.88, 1.41)	
Recency of injecting	>12 months	251 (14%)	26 (10%)	‐ref‐	‐ref‐
	Within 1–12 months	363 (21%)	69 (19%)	2.03 (1.25. 3.29)	1.94 (1.18, 3.18)
	Within last month, <daily	620 (35%)	122 (20%)	2.12 (1.34, 3.33)	1.91 (1.20, 3.04)
	Within last month, ≥daily	535 (30%)	147 (28%)	3.28 (2.09, 2.13)	3.09 (1.93, 4.91)
Main drug injected in last month	None	614 (35%)	95 (15%)	‐ref‐	
	Heroin	410 (23%)	85 (21%)	1.42 (1.04, 1.97)	
	Other opioids	162 (9%)	52 (32%)	2.58 (1.78, 3.83)	
	Methamphetamine	557 (31%)	126 (23%)	1.60 (1.19, 2.15)	
	Other	26 (1%)	6 (23%)	1.69 (0.64, 4.19)	
Recruitment wave	Wave 1 (2018–2019)	1064 (60%)	253 (24%)	‐ref‐	‐ref‐
	Wave 2 (2019–2021)	705 (40%)	111 (16%)	0.60 (0.47, 0.77)	0.55 (0.43, 0.72)

Abbreviations: aOR, adjusted odds ratio; CI, confidence interval; HCV, hepatitis C virus; OAT, opioid agonist treatment; OR, odds ratio; RNA, ribonucleic acid.

^a^ With valid HCV RNA point‐of‐care test results, *N* = 1769.

^b^ Other refers to any individual who did not identify as a man, woman or as transgender.

^c^ Not reported transgender/other.

### 3.5 HCV treatment

Among Aboriginal participants with evidence of current or past HCV infection (*n* = 278), 65% (*n* = 181) reported ever receiving HCV treatment, increasing from 63% (110/175) during 2018–2019 to 69% (71/103) during 2019–2021 (*p* = 0.30) (Figure 1b). Among non‐Aboriginal participants with evidence of current or past HCV infection (*n* = 972), 70% (*n* = 681) reported HCV treatment, increasing from 67% (410/613) during 2018–2019 to 75% (271/359) during 2019–2021 (*p* < 0.001) (Figure 1b). The vast majority of those treated received DAA therapy, including 92% (167/181) and 86% (585/681) of Aboriginal and non‐Aboriginal participants, respectively.

### 3.6 Factors associated with HCV treatment among Aboriginal and Torres Strait Islander participants

**FIGURE 3:**
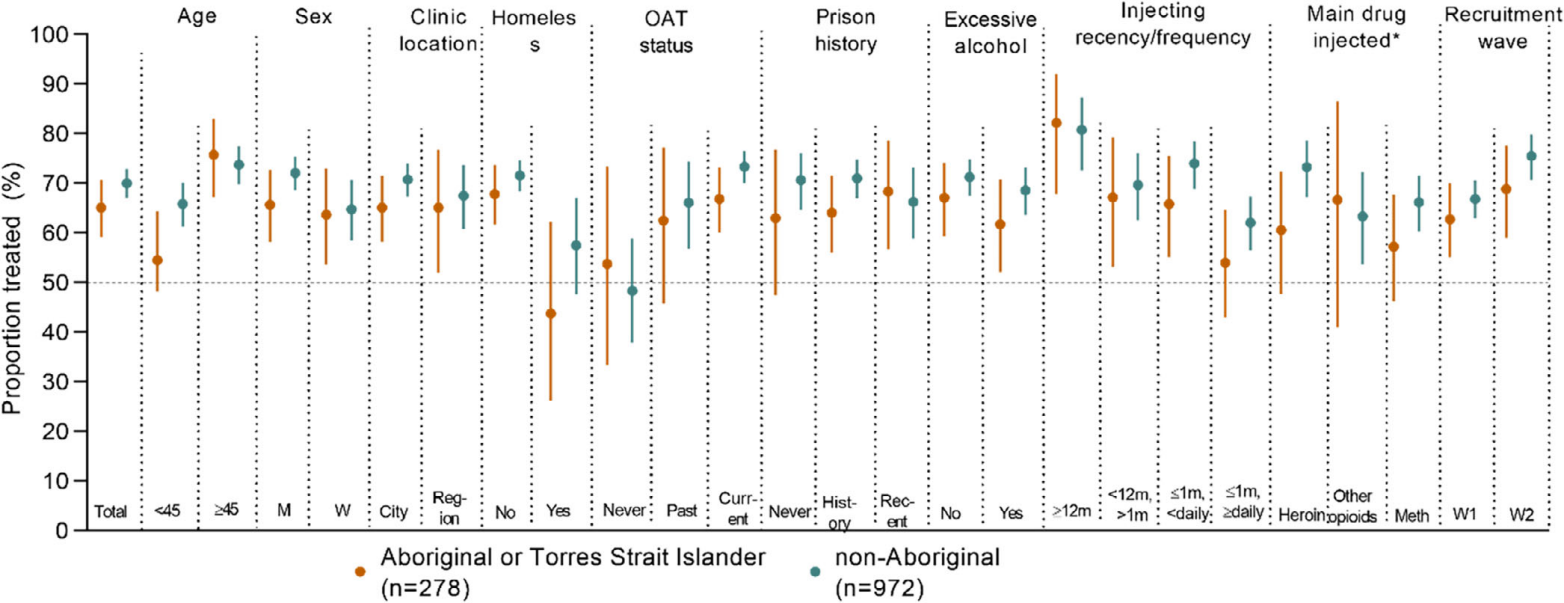
Self‐reported hepatitis C virus (HCV) treatment** reported among ETHOS Engage participants who had current or previous chronic HCV infection, by Aboriginal or Torres Strait Islander status. *Main drug injected in last month; “other” not shown due to small numbers. **The proportion of participants with self‐reported treatment is represented by the dot; the bar represents the 95% confidence interval around each proportion. OAT, opioid agonist treatment.

HCV treatment by characteristics of interest among Aboriginal participants is presented in Table 4 and Figure 3. In adjusted analyses among Aboriginal participants, recruitment wave was not associated with HCV treatment. In adjusted analyses, older age was associated with higher treatment uptake (vs. <45; ≥45, aOR 2.46, 95% CI 1.38, 4.35), whereas HCV treatment was lower among Aboriginal participants who were homeless (aOR 0.40, 95% CI 0.18, 0.92) and among those who had injected drugs ≥daily (vs. injected more than 1 year ago, aOR: 0.28, 95% CI 0.11, 0.72) (Table 4). Similarly, among Aboriginal participants who injected drugs in the previous month, younger age and homelessness were associated with lower odds of HCV treatment (Table S5).

**TABLE 4:** Factors associated with HCV treatment among Aboriginal and Torres Strait Islander participants with past or current HCV infection.

Characteristic		Previous or current HCV infection, *n* (col%)	History of HCV treatment, *n* (row%)	OR (95% CI)	aOR (95% CI)
Total		278	181 (65%)		
Age at enrolment	<45	154 (55%)	87 (56%)	‐ref‐	‐ref‐
	≥45	124 (45%)	94 (76%)	2.41 (1.43, 4.06)	2.46 (1.38, 4.35)
Gender	Man	175 (63%)	115 (65%)	‐ref‐	‐ref‐
	Woman	102 (37%)	65 (64%)	0.92 (0.55, 1.52)	1.27 (0.71, 2.27)
	Transgender/other^a^	1 (<1%)	1 (100%)	Omitted	Omitted
Location of clinic	Major cities of Australia	215 (77%)	140 (65%)	‐ref‐	
	Regional Australia	63 (23%)	41 (65%)	1.00 (0.55, 1.80)	
Homeless	No	246 (89%)	167 (68%)	‐ref‐	‐ref‐
	Yes	32 (12%)	14 (44%)	0.40 (0.17, 0.78)	0.40 (0.18, 0.92)
OAT status	Never	26 (9%)	14 (54%)	‐ref‐	‐ref‐
	Past	40 (14%)	25 (62%)	1.43 (0.52, 3.89)	1.42 (0.48, 4.17)
	Current	212 (76%)	142 (67%)	1.74 (0.76, 3.95)	1.34 (0.55, 3.29)
Incarceration history	Never	46 (17%)	29 (63%)	‐ref‐	‐ref‐
	History only	156 (56%)	100 (64%)	1.05 (0.53, 2.08)	1.12 (0.53, 2.35)
	Recent	76 (27%)	52 (68%)	1.27 (0.59, 2.74)	1.94 (0.81, 4.68)
Excessive alcohol consumption^b^	No	167 (60%)	112 (67%)	‐ref‐	
	Yes	110 (40%)	68 (62%)	0.80 (0.48, 1.31)	
Recency of injecting	>12 months	45 (16%)	37 (82%)	‐ref‐	‐ref‐
	Within 1–12 months	55 (20%)	37 (67%)	0.44 (0.17, 1.15)	0.52 (0.19, 1.41)
	Within last month, <daily	91 (33%)	60 (66%)	0.42 (0.17, 1.01)	0.42 (0.16, 1.06)
	Within last month, ≥daily	87 (31%)	47 (54%)	0.25 (0.11, 0.61)	0.28 (0.11, 0.72)
Main drug injected in last month	None	100 (36%)	74 (74%)	‐ref‐	
	Heroin	66 (%)	40 (61%)	0.54 (0.28, 1.05)	
	Other opioids	18 (24%)	12 (67%)	0.70 (0.24, 2.06)	
	Methamphetamine	89 (32%)	51 (57%)	0.47 (0.26, 0.87)	
	Other	5 (2%)	4 (80%)	1.41 (0.15, 13.15)	
Recruitment wave	Wave 1 (2018–2019)	175 (63%)	110 (63%)	‐ref‐	‐ref‐
	Wave 2 (2019–2021)	103 (37%)	71 (69%)	1.31 (0.78, 2.20)	1.56 (0.88, 2.73)

Abbreviations: aOR, adjusted odds ratio; CI, confidence interval; HCV, hepatitis C virus; OAT, opioid agonist treatment; OR, odds ratio.

^a^ Other refers to any individual who did not identify as a man, woman or as transgender.

^b^ Not reported transgender/other.

### 3.7 Factors associated with HCV treatment among non‐Aboriginal participants

HCV treatment by characteristics of interest among non‐Aboriginal participants is presented in Table 5 and Figure 3. In adjusted analyses, non‐Aboriginal participants recruited between 2019 and 2021 (wave 2) were more likely to have received HCV treatment (vs. 2018–2019 [wave 1] aOR: 1.45, 95% CI 1.21, 2.23) (Table 5). In adjusted analyses, a history of and current OAT was also associated with higher HCV treatment (history vs. never receiving OAT, past OAT aOR: 2.03, 95% CI 1.14, 3.59; current vs. never receiving OAT, aOR: 2.73, 95% CI 1.71, 4.53), whereas HCV treatment was lower among non‐Aboriginal participants who were women (aOR: 0.65, 95% CI 0.46, 0.90), those who were homeless (aOR: 0.64, 95% CI 0.41, 0.99) and those who had injected drugs ≥daily (vs. injected more than 1 year ago aOR: 0.52, 95% CI 0.30, 0.88) (Table 5).

**TABLE 5:** Factors associated with HCV treatment among non‐Aboriginal participants with past or current HCV infection.

Characteristic		Previous or current HCV infection, *n* (col%)	History of HCV treatment, *n* (row%)	OR (95% CI)	aOR (95% CI)
Total		972	681 (70%)		
Age at enrolment	<45	464 (48%)	306 (66%)	‐ref‐	‐ref‐
	≥45	508 (52%)	375 (74%)	1.46 (1.11, 1.92)	1.33 (0.99, 1.78)
Gender	Man	715 (74%)	516 (72%)	‐ref‐	‐ref‐
	Woman	250 (26%)	162 (65%)	0.71 (0.52, 0.96)	0.65 (0.46, 0.90)
	Transgender/other^a^	7 (1%)	3 (43%)	0.29 (0.06, 1.30)	Omitted
Location of clinic	Major cities of Australia	753 (78%)	533 (71%)	‐ref‐	
	Regional Australia	219 (22%)	148 (68%)	0.86 (0.62, 1.19)	
Homeless	No	866 (89%)	620 (72%)	‐ref‐	‐ref‐
	Yes	106 (11%)	61 (58%)	0.54 (0.36, 0.81)	0.64 (0.41, 0.99)
OAT status	Never	95 (10%)	46 (48%)	‐ref‐	‐ref‐
	Past	121 (12%)	80 (66%)	2.08 (1.20, 3.60)	2.03 (1.14, 3.59)
	Current	756 (78%)	555 (73%)	2.94 (1.91, 4.54)	2.73 (1.71, 4.35)
Incarceration history	Never	259 (27%)	183 (71%)	‐ref‐	‐ref‐
	History only	535 (55%)	380 (71%)	1.02 (0.73, 1.41)	0.87 (0.61, 1.23)
	Recent	178 (18%)	118 (66%)	0.82 (0.54, 1.23)	0.73 (0.46, 1.15)
Excessive alcohol consumption^b^	No	596 (61%)	425 (71%)	‐ref‐	
	Yes	369 (38%)	252 (69%)	0.88 (0.66, 1.16)	
Recency of injecting	>12 months	120 (12%)	97 (81%)	‐ref‐	‐ref‐
	Within 1–12 months	188 (19%)	131 (70%)	0.54 (0.31, 0.95)	0.62 (0.36, 1.12)
	Within last month, <daily	345 (36%)	255 (74%)	0.67 (0.40, 1.12)	0.86 (0.50, 1.47)
	Within last month, ≥daily	319 (33%)	198 (62%)	0.39 (0.23, 0.64)	0.52 (0.30, 0.88)
Main drug injected in last month	None	308 (32%)	228 (74%)	‐ref‐	
	Heroin	247 (25%)	181 (73%)	0.96 (0.66, 1.40)	
	Other opioids	112 (12%)	71 (63%)	0.61 (0.38, 0.96)	
	Methamphetamine	290 (30%)	192 (66%)	0.69 (0.48, 0.98)	
	Other	15 (2%)	9 (60%)	0.53 (0.18, 1.52)	
Recruitment wave	Wave 1 (2018–2019)	613 (63%)	410 (67%)	‐ref‐	‐ref‐
	Wave 2 (2019–2021)	359 (37%)	271 (75%)	1.52 (1.14, 2.04)	1.45 (1.21, 2.23)

Abbreviations: aOR, adjusted odds ratio; CI, confidence interval; HCV, hepatitis C virus; OAT, opioid agonist treatment; OR, odds ratio.

^a^ Other refers to any individual who did not identify as a man, woman or as transgender.

^b^ Not reported transgender/other.

## 4 DISCUSSION

Between 2018 and 2021, the burden of HCV infection among PWID declined in this national cohort, but progress towards HCV elimination was uneven and risks widening health disparities between Aboriginal peoples and other Australians. Significant changes in current HCV infection and treatment uptake among non‐Aboriginal PWID were not mirrored among Aboriginal PWID. Population characteristics typically associated with greater vulnerability and marginalisation, including recent injecting drug use and homelessness, were positively associated with current HCV infection, and negatively associated with treatment uptake. Focused clinical interventions, targeted health policy, community‐led and culturally appropriate responses are required to ensure equitable health outcomes among Aboriginal PWID as Australia moves towards HCV elimination.

During the study period, a significant decrease in current HCV infection was seen among non‐Aboriginal PWID, but not Aboriginal PWID. In 2018–2019, prevalence of current HCV infection was 24% in both populations. In 2019–2021, an 8% absolute decline in current HCV infection prevalence was seen among non‐Aboriginal PWID compared with a 3% absolute decline among Aboriginal PWID (falling to 16% compared with 21%). Higher prevalence of current HCV infection among Aboriginal PWID has been documented in other cohort studies [10, 22, 25]. Despite being recognised as a priority population, Aboriginal PWID continue to be disproportionately impacted by HCV infection [5].

Cumulative HCV treatment uptake increased over time, however, Aboriginal PWID were less likely to have reported receiving treatment than non‐Aboriginal PWID. Historically, HCV treatment uptake among Aboriginal peoples, including PWID, has been lower than non‐Aboriginal people in both the interferon and DAA eras [8, 9, 26]. Disparities in HCV treatment uptake among Aboriginal and some First Nations peoples internationally (e.g., Canada) have persisted despite government subsidised DAA access [8, 9, 26, 27]. Systemic and racially driven disadvantages faced by Aboriginal and other First Nations peoples, including transgenerational trauma and historical exclusion from health care, are magnified among PWID, layered with additional concerns of shame and stigma associated with injecting drug use and HCV infection [15, 17, 28, 29]. To address this, community‐based health services that provide culturally appropriate tailored models of care and treatment delivery for Aboriginal PWID may encourage health seeking behaviours and continued health‐care engagement [15, 16, 28, 29, 30, 31].

Among Aboriginal PWID, current OAT was positively associated with current HCV infection, a result that was not found in non‐Aboriginal participants. Among non‐Aboriginal PWID, current OAT was positively associated with self‐reported HCV treatment, a result that was not found in Aboriginal participants. Opioid agonist therapy has been shown to increase linkage to HCV care, including HCV treatment among PWID [32]; however, the integration of HCV services within the OAT may not be sufficient to engage Aboriginal clients, and optimising this setting to facilitate treatment uptake and reduce current infection among Aboriginal PWID may require the input and expertise of Aboriginal health workers and peers.

Current injecting drug use was positively associated with current HCV infection and negatively associated with treatment uptake. Scale‐up of HCV testing and treatment among people who report recent (and particularly, frequent) injecting, in combination with harm reduction, is critical for HCV elimination. A higher prevalence of injecting behaviours associated with HCV acquisition among Aboriginal PWID have been reported, including receptive needle sharing, frequent injecting, younger age at first injecting, and injecting while incarcerated [12, 13, 21, 23]. Development and expansion of culturally safe harm reduction strategies for Aboriginal PWID, involving Aboriginal peer and health workers, may assist in HCV care and prevention [5, 33].

Homelessness was associated with higher prevalence of current HCV infection and lower treatment among Aboriginal and non‐Aboriginal PWID. The many potential barriers to engaging and retaining people who are homeless or unstably housed in health care have been well documented, including competing priorities multiple psychosocial factors and stigma [34, 35]. While managing HCV in this context is challenging, some success has been demonstrated with innovative models, including provision of holistic care in shelters or locations frequented by people who are homeless [34, 36]. Further, unstable housing is associated with HCV transmission among PWID highlighting the need for interventions among this population to mitigate housing instability and reduce the risk of HCV acquisition [37].

Non‐Aboriginal women had lower self‐reported treatment compared to non‐Aboriginal men. Although this was not a result found among Aboriginal women, more evidence of the gender‐specific factors influencing HCV care is critical [38]. Women who inject drugs have been shown to have lower treatment uptake than men [8, 18, 39, 40], with emerging evidence that younger age, multiple records of childbirth, and delivering in the DAA treatment era are associated with lower treatment uptake [41]. Five participants in ETHOS Engage wave 2 indicated that they were or might be pregnant, one of which was ever eligible for treatment (data not shown). While these numbers are small and do not indicate lifetime experience of pregnancy, greater understanding of the factors associated with treatment uptake among women who inject drugs should be a priority. The evaluation of the gender‐specific factors associated with the HCV cascade of care is the focus of a subsequent paper.

Although attempts were made to diminish bias and control for confounding, this analysis has limitations [18, 19]. Participants were opportunistically recruited in health‐care settings, which may introduce selection bias, overestimate treatment uptake, and underestimate HCV RNA prevalence in comparison with the broader PWID population. Data obtained through questionnaires relied on participant recall and self‐report. While self‐report is considered a reliable source of data collection among PWID, recall bias could not be systematically alleviated. However, social‐desirability bias was reduced through questionnaire self‐administration. Furthermore, our study focuses on current HCV infection and self‐reported treatment; however, other outcomes along the cascade of care, including testing, treatment outcomes and reinfection should be further assessed. In addition to reduced number of sites, recruitment for wave 2 appears to be over a longer period than wave 1; however, this is due to a 6‐month study suspension (March–September 2020) due to COVID‐19 prevention and control measures. While we have seen a reduction in HCV prevalence and an increase in self‐reported treatment, the COVID‐19 pandemic has likely affected the health and wellbeing of PWID globally [42] and this recruitment occurred during a time of potentially delayed HCV elimination efforts [43]. Additionally, wave 2 recruitment occurred during a time of decreased in‐person/daily dosing for OAT as the number of takeaway OAT doses increased to reduce foot traffic in clinics [44, 45]. This would have impacted our opportunistic recruitment strategy as recruitment in sites was slightly lower for wave 2 than it was for wave 1, although the demographic and behavioural characteristics between recruitment waves were similar. ETHOS Engage wave 3 recruitment is underway, and the results obtained from this will be useful in determining if these results are sustained beyond the initial COVID‐19 lockdowns. Finally, the multiple comparisons made in our results may have led to an increased risk of type I error.

Success in the management of HCV infection and other infectious diseases should guide clinical and public health policy to increase DAA uptake and reduce HCV RNA prevalence among Aboriginal PWID. For HCV, interventions involving simplified testing strategies (including point‐of‐care), patient and provider education, and care coordination have improved testing, linkage to care and treatment uptake [16, 46, 47]. For other infectious diseases (including COVID‐19), testing and management among Indigenous and First Nations peoples has been facilitated by novel testing strategies (point‐of‐care, self‐testing), targeted health initiatives, health‐care worker cultural competency, and leadership and engagement of the Aboriginal community in decisions about their health care [46, 48, 49]. A multifaceted approach involving community leadership, integration of HCV care and education (including peer‐to‐peer knowledge exchange), and social support within services frequented by Aboriginal PWID is recommended to improve DAA uptake and health outcomes [31, 33, 50].

Australia has made significant progress towards national HCV elimination targets, however, progress among Aboriginal peoples, including PWID, has been more limited. Identifying those most at‐risk and working with affected communities will be critical in coordinating culturally safe clinical and public health responses to ensure no one is left behind. Sustained DAA uptake and equitable access to HCV care, treatment and prevention must be assured if HCV elimination is to be achieved. To ensure the effectiveness, impact and cultural appropriateness of viral hepatitis elimination strategies, it is important to involve Aboriginal PWID in decision making, policy development, care delivery and setting research agendas.

## AUTHOR CONTRIBUTIONS

Jason Grebely, Gregory J. Dore and Carla Treloar conceived and designed the ETHOS Engage study, including the participant questionnaire. Steven Hobday, Heather Valerio, Jason Grebely and Marianne Martinello contributed to these research aims, analysis and interpretation of the results. Steven Hobday and Heather Valerio wrote the first draft of the manuscript under the supervision of Marianne Martinello. All authors contributed to the critical review and development of this final manuscript for publication.

## CONFLICT OF INTEREST STATEMENT

Cepheid and Merck Sharp and Dohme Corp were not involved in the study design, methodology and writing of this manuscript. The opinions expressed in the paper are those of the authors and do not necessarily represent those of Cepheid or Merck Sharp and Dohme Corp. The views expressed in this manuscript do not necessarily represent the position of the Australian Government. The content is solely the responsibility of the authors. None of the authors had commercial relationships that might pose a conflict of interest in connection with this manuscript. Phillip Read has received research grant funding from Gilead Sciences. Charles Henderson has received research grant funding from Indivior. Louisa Degenhardt has received investigator‐initiated untied educational grants for studies of opioid medications in Australia from Indivior, Mundipharma and Seqirus. Gregory J. Dore has received research grants from Gilead and Abbvie. Jason Grebely is a consultant/advisor and has received research grants from Abbvie, Biolytical, Camurus, Cepheid, Gilead Sciences, Hologic, Indivior and Merck/MSD. All remaining authors have no potential conflicts to declare.

## FUNDING INFORMATION

The Enhancing Treatment of Hepatitis C in Opioid Substitution Settings (ETHOS) Engage study is funded by a National Health and Medical Research Council (NHMRC) Partnership project grant (1103165), including funding from New South Wales Health and Cepheid. This study was also supported in part by research grants from Investigator‐Initiated Studies Program of Merck Sharp and Dohme Corp and the Australian Department of Health. The Kirby Institute is also funded by the Australian Department of Health. Jason Grebely is supported by an NHMRC Investigator Grant (1176131). Gregory J. Dore is supported by an NHMRC Practitioner Fellowship and Investigator Grant (2008276). Louisa Degenhardt is supported by a National Institute of Health National Institute on Drug Abuse grant (R01DA1104470). Marianne Martinello is supported by an NHMRC Early Career Fellowship (11568245).

## ETHICS STATEMENT

This work was approved by the following ethics committees: Aboriginal Health and Medical Research Council (AHMRC Ref: 1279/17); Human Research Ethics Committee at St Vincent's Hospital Sydney (HREC Ref: HREC/17/SVH/113).

## Supporting information


**Data S1:** Supporting Information.

## Endnote

* Aboriginal and Torres Strait Islander Peoples are collective terms to describe diverse groups of peoples with unique cultures, languages, customs and ways of life. Aboriginal and Torres Strait Islander Peoples are the First Peoples of country now known as Australia and have an ongoing connection to land, skies and waterways. We will be respectfully using the term Aboriginal Peoples to refer to these groups.
