# Supplementary material for: Evaluating the prevalence of current hepatitis C infection and treatment among Aboriginal and Torres Strait Islander peoples who inject drugs in Australia: The ETHOS engage study
Source: Drug Alcohol Rev. 2023 Aug 9;42(7):1617–32. doi: 10.1111/dar.13723 (PMC10946462; doi:10.1111/dar.13723)
Supplement: Supplementary file 1 — Data S1: Supporting Information. [file DAR-42-1617-s001.docx]

**Table S1.** ETHOS Engage recruitment sites, dates, and Aboriginal and Torres Strait Islander participation, by recruitment wave

| **Site number** | **State** | **Site location** | **Main function of site** | **Wave 1 recruitment** | **Wave ,1  N (col %)** | **Wave 2 recruitment** | **Wave 2,  N (col %)** | **Time between recruitment waves (mo*)** |
| --- | --- | --- | --- | --- | --- | --- | --- | --- |
| 1 | NSW | MC | OAT | 28-31 May 2018 | 16 (5%) | 1, 3-5 Feb 2021 | 17 (8%) | 33 |
| 2 | NSW | MC | DAS | 18-21 Jun 2018 | 29 (9%) | 15-18 Mar 2021 | 11 (5%) | 33 |
| 3 | NSW | RA | OAT | 22-30 Jun 2018 | 13 (4%) | 21 June 2021 | 1 (<0.5%) | 36 |
| 4 | Qld | RA | NSP | 16-19 Jul 2018 | 18 (5%) | 29 Mar-1 Apr 2021 | 14 (6%) | 33 |
| 5 | NSW | MC | OAT | 20-22 Aug 2018 | 10 (3%) | 13-15 Jan 2020 | 12 (6%) | 17 |
| 6 | NSW | MC | OAT | 12-19 Sept 2018 | 29 (9%) | 20-21 Jan 2020 | 17 (8%) | 17 |
|  |  |  |  |  |  | 3 –4 Feb 2020 |  |  |
| 7 | Qld | MC | NSP | 5-8 Nov 2018 | 21 (6%) | 11-14 Nov 2019 | 26 (12%) | 12 |
| 8 | NSW | RA | OAT | 21-23 Nov 2018 | 5 (1%) |  | | |
| 9 | NSW | MC | OAT | 26-29 Nov 2018 | 28 (8%) | 9-12 Dec 2019 | 17 (8%) | 12 |
| 10 | NSW | MC | OAT | 22 Jan 2019 | 8 (2%) | 10-11 Mar 2020 | 12 (6%) | 13 |
|  |  |  |  | 5 Feb 2019 |  | 4 –5 Mar 2021 |  |  |
|  |  |  |  | 5 Mar 2019 |  |  |  |  |
| 11 | NSW | RA | OAT | 11-12 Feb 2019 | 33 (10%) | 28-29 Sept 2020 | 12 (6%) | 20 |
| 12 | NSW | RA | OAT | 13-15 Feb 2019 | 8 (2%) | 30 Sept-2 Oct 2020 | 3 (1%) | 20 |
| 13 | NSW | RA | OAT | 20-22 Feb 2019 | 18 (5%) | 6-9 Oct 2020 | 2 (1%) | 20 |
| 14 | SA | MC | OAT | 12-13 Mar 2019 | 7 (2%) | 31 May-1 Jun 2021 | 6 (3%) | 27 |
| 15 | SA | MC | OAT | 14-15 Mar 2019 | 7 (2%) | 2-3 Jun 2021 | 5 (2%) | 27 |
| 16 | NSW | RA | OAT | 25-27 Mar 2019 | 11 (3%) | 10-12 Nov 2020 | 9 (4%) | 20 |
| 17 | NSW | MC | OAT | 13-16 May 2019 | 14 (4%) | 17-20 Nov 2020 | 15 (7%) | 18 |
| 18 | NSW | MC | OAT | 20-23 May 2019 | 36 (11%) | 26-29 Oct 2020 | 16 (7%) | 18 |
| 19 | WA | MC | DAS | 17-19 Jun 2019 | 11 (3%) | 19-21 May 2021 | 13 (6%) | 23 |
| 20 | WA | MC | DAS | 20-21 Jun 2019 | 1 (<0.5%) | 17-18 May 2021 | 4 (2%) | 23 |
| 21 | NSW | MC | OAT | 27-28 Jun 2019 | 1 (<0.5%) |  | | |
| 22 | Qld | RA | NSP | 22-23 Jul 2019 | 0 (0%) |  | | |
| 23 | Qld | RA | NSP | 24-25 Jul 2019 | 6 (2%) |  | | |
| 24 | NSW | MC | OAT | 3-6 Sept 2019 | 4 (1%) | 12-15 Apr 2021 | 4 (2%) | 20 |
| 25 | NSW | MC | OAT | 25-27 Sept 2019 | 3 (1%) | 22-25 Feb 2021 | 2 (1%) | 18 |

*****time calculated as the number of months from first recruitment date in Wave 1 to first recruitment in Wave 2.

DAS, drug and alcohol service; MC, major city of Australia; NSP, needle and syringe program; NSW, New South Wales; OAT, opioid agonist therapy; Qld, Queensland; RA, regional Australia; SA, South Australia; WA, Western Australia.

**Table S2.** Characteristics of Aboriginal and Torres Strait Islander participants, by recruitment wave

|  | | **Overall population** | **Wave 1 (2018-2019)** | **Wave 2 (2019-2021)** | **Participants enrolled in both Wave 1 and Wave 2** | **Participants only enrolled in Wave 2** |
| --- | --- | --- | --- | --- | --- | --- |
| Total | | 555 | 337 | 290 | 72 | 218 |
| Median age (interquartile range) | | 42 (36, 48) | 42 (36, 48) | 43 (37, 50) | 47 (40, 51) | 42 (37, 50) |
| Gender | Man | 324 (58%) | 190 (56%) | 173 (60%) | 39 (54%) | 134 (61%) |
|  | Woman | 229 (41%) | 147 (44%) | 115(40%) | 33 (46%) | 82 (38%) |
|  | Transgender/other^*^ | 2 (<1%) | 0 (0%) | 2 (1%) | 0 (0%) | 2 (1%) |
| Location of clinic | Major cities of Australia | 402 (72%) | 225 (67%) | 235 (81%) | 58 (81%) | 177 (81%) |
|  | Regional Australia | 153 (28%) | 112 (33%) | 55 (19%) | 14 (19%) | 41 (19%) |
| Homeless | No | 485 (87%) | 293 (87%) | 261 (90%) | 69 (96%) | 192 (88%) |
|  | Yes | 70 (13%) | 44 (13%) | 29 (10%) | 3 (4%) | 26 (12%) |
| Opioid agonist treatment status | Never | 100 (18%) | 61 (18%) | 40 (14%) | 1 (1%) | 39 (18%) |
|  | Past | 66 (12%) | 35 (10%) | 36 (12%) | 5 (7%) | 31 (14%) |
|  | Current | 389 (70%) | 241 (72%) | 214 74%) | 66 (92%) | 148 (68%) |
| Incarceration history | Never | 120 (22%) | 74 (22%) | 59 (20%) | 13 (18%) | 46 (21%) |
|  | History only | 600 (54%) | 182 (54%) | 161 (56%) | 43 (60%) | 118 (54%) |
|  | Recent | 135 (24%) | 81 (24%) | 70 (24%) | 16 (22%) | 54 (25%) |
| Excessive alcohol consumption* | No | 339 (61%) | 219 (65%) | 173 (60%) | 53 (74%) | 120 (56%) |
|  | Yes | 214 (39%) | 118 (35%) | 115 (40%) | 19 (26%) | 96 (44%) |
| Recency of injecting | >12 months | 76 (14%) | 52 (15%) | 39 (13%) | 15 (21%) | 24 (11%) |
|  | Within 1-12 months | 130 (23%) | 78 (23%) | 67 (23%) | 15 (21%) | 52 (24%) |
|  | Within last month, <daily | 177 (32%) | 104 (31%) | 91 (31%) | 18 (25%) | 73 (33%) |
|  | Within last month, ≥daily | 172 (31%) | 103 (31%) | 93 (32%) | 24 (33%) | 69 (32%) |
| Main drug injected in last month | None | 206 (37%) | 130 (39%) | 106 (37%) | 30 (42%) | 76 (35%) |
|  | Heroin | 180 (19%) | 57 (17%) | 70 (24%) | 19 (27%) | 51 (223%) |
|  | Other opioids | 29 (5%) | 17 (5%) | 15 (5%) | 3 (4%) | 12 (6%) |
|  | Methamphetamine | 200 (36%) | 123 (37%) | 97 (33%) | 20 (28%) | 77 (35%) |
|  | Other | 12 (2%) | 10 (3%) | 2 (1%) | 0 (0%) | 2 (1%) |

All % are column proportions; *other refers to any individual who did not identify as a man, woman, or as transgender; **not reported transgender/other.

**Table S3.** Characteristics of non-Aboriginal participants, by recruitment wave

|  | | **Overall population** | **Wave 1 (2018-2019)** | **Wave 2 (2019-2021)** | **Participants enrolled in both Wave 1 and Wave 2** | **Participants only enrolled in Wave 2** |
| --- | --- | --- | --- | --- | --- | --- |
| Total | | 1,840 | 1,106 | 921 | 187 | 734 |
| Median age (interquartile range) | | 44 (37, 51) | 44 (37, 51) | 44 (38, 51) | 47 (40, 55) | 44 (38, 51) |
| Gender | Man | 1267 (69%) | 742 (67%) | 647 (70%) | 122 (65%) | 525 (72%) |
|  | Woman | 557 (30%) | 361 (32%) | 258 (28%) | 62 (33%) | 196 (26%) |
|  | Transgender/other^*^ | 16 (1%) | 3 (<1%) | 16 (2%) | 3 (2%) | 13 (2%) |
| Location of clinic | Major cities of Australia | 1414 (77%) | 818 (74%) | 725 (82%) | 156 (83%) | 596 (81%) |
|  | Regional Australia | 426 (23%) | 288 (26%) | 169 (18%) | 31 (17%) | 138 (19%) |
| Homeless | No | 1649 (90%) | 992 (90%) | 838 (91%) | 181 (97%) | 657 (90%) |
|  | Yes | 191 (10%) | 114 (10%) | 83 (9%) | 6 (3%) | 77 (10%) |
| Opioid agonist treatment status | Never | 271 (15%) | 114 (13%) | 132 (14%) | 5 (3%) | 127 (17%) |
|  | Past | 239 (13%) | 133 (12%) | 137 (15%) | 31 (17%) | 106 (14%) |
|  | Current | 1330 (72%) | 829 (75%) | 652 (71%) | 151 (81%) | 501 (68%) |
| Incarceration history | Never | 651 (35%) | 395 (36%) | 305 (33%) | 49 (26%) | 256 (35%) |
|  | History only | 881 (48%) | 533 (48%) | 471 (51%) | 123 (66%) | 348 (47%) |
|  | Recent | 308 (17%) | 178 (16%) | 145 (16%) | 15 (8%) | 130 (18%) |
| Excessive alcohol consumption** | No | 1117 (61%) | 660 (60%) | 578 (64%) | 121 (66%) | 457 (63%) |
|  | Yes | 707 (39%) | 443 (40%) | 327 (36%) | 63 (34%) | 264 (37%) |
| Recency of injecting | >12 months | 258 (14%) | 163 (15%) | 135 (15%) | 40 (21%) | 95 (13%) |
|  | Within 1-12 months | 376 (20%) | 229 (21%) | 193 (21%) | 46 (25%) | 147 (20%) |
|  | Within last month, <daily | 645 (35%) | 390 (35%) | 313 (34%) | 58 (31%) | 255 (35%) |
|  | Within last month, ≥daily | 561 (30%) | 324 (29%) | 280 (30%) | 43 (23%) | 237 (32%) |
| Main drug injected in last month | None | 634 (34%) | 392 (35%) | 328 (36%) | 86 (46%) | 242 (33%) |
|  | Heroin | 427 (23%) | 255 (23%) | 212 (23%) | 10 (21%) | 172 (23%) |
|  | Other opioids | 172 (9%) | 115 (10%) | 70 (8%) | 13 (7%) | 57 (8%) |
|  | Methamphetamine | 580 (32%) | 327 (30%) | 299 (32%) | 46 (25%) | 253 (34%) |
|  | Other | 27 (1%) | 17 (2%) | 12 (1%) | 2 (1%) | 10 (1%) |

All % are column proportions; *other refers to any individual who did not identify as a man, woman, or as transgender; **not reported transgender/other**.**

**Table S4.** Factors associated with current HCV infection among Aboriginal and Torres Strait Islander participants who had injected in the previous month with valid HCV RNA point-of-care test results (N=337)

| **Characteristic** | | **Total known HCV RNA result, n (col%)** | **Current HCV RNA infection, n(row%)** | **OR (95% CI)** | **aOR (95% CI)** |
| --- | --- | --- | --- | --- | --- |
| **Total** | | 337 | 88 (26%) |  | |
| Age at enrolment | <45 | 210 (62%) | 59 (28%) | -ref- | -ref- |
|  | ≥45 | 127 (38%) | 29 (23%) | 0.76 (0.45, 1.26) | 0.72 (0.42, 1.24) |
| Gender | Man | 196 (58%) | 53 (27%) | -ref- | -ref- |
|  | Woman | 140 (42%) | 35 (25%) | 0.90 (0.55, 1.48) | 0.53 (0.48, 1.41) |
|  | Transgender/other^*^ | 1 (<1%) | 0 (0%) | omitted | omitted |
| Location of clinic | Major cities of Australia | 251 (74%) | 67 (27%) | -ref- |  |
|  | Regional Australia | 86 (26%) | 21 (24%) | 0.89 (0.50, 1.56) |  |
| Homeless | No | 283 (84%) | 70 (25%) | -ref- | -ref- |
|  | Yes | 54 (16%) | 18 (33%) | 1.52 (0.81, 2.85) | 1.74 (0.88, 3.44) |
| Opioid agonist treatment status | Never | 71 (21%) | 11 (15%) | -ref- | -ref- |
|  | Past | 54 (16%) | 17 (31%) | 2.51 (1.06, 5.93) | 2.32 (0.95, 5.68) |
|  | Current | 212 (63%) | 60 (28%) | 2.15 (1.06, 4.37) | 2.01 (0.94, 4.31) |
| Incarceration history | Never | 72 (21%) | 14 (19%) | -ref- | -ref- |
|  | History only | 176 (52%) | 48 (27%) | 1.56 (0.79, 3.04) | 1.45 (0.72, 2.96) |
|  | Recent | 89 (26%) | 26 (29%) | 1.71 (0.81, 3.59) | 1.32 (0.59, 2.93) |
| Excessive alcohol consumption** | No | 193 (57%) | 47 (24%) | -ref- |  |
|  | Yes | 143 (42%) | 41 (29%) | 1.25 (0.76, 2.04) |  |
| Frequency of injecting | Within last month, <daily | 174 (52%) | 43 (25%) | -ref- | -ref- |
|  | Within last month, ≥daily | 163 (48%) | 45 (28%) | 1.16 (0.71, 1.89) | 0.92 (0.54, 1.57) |
| Main drug injected in last month | Heroin | 103 (31%) | 33 (32%) | -ref- | -ref- |
|  | Other opioids | 28 (8%) | 6 (21%) | 0.58 (0.21, 1.56) | 0.62 (0.22, 1.70) |
|  | Methamphetamine | 195 (58%) | 47 (24%) | 0.67 (0.40, 1.14) | 0.75 (0.42, 1.34) |
|  | Other | 11 (3%) | 2 (18%) | 0.47 (0.09, 2.30) | 0.47 (0.09, 2.42) |
| Recruitment wave | Wave 1 (2018-2019) | 197 (58%) | 52 (26%) | -ref- | -ref- |
|  | Wave 2 (2019-2021) | 140 (41%) | 36 (26%) | 0.97 (0.59, 1.60) | 0.93 (0.56, 1.54) |

*other refers to any individual who did not identify as a man, woman, or as transgender; **not reported transgender/other.

aOR, adjusted odds ratio; CI, confidence interval; HCV, hepatitis C virus; OR, odds ratio; RNA, ribonucleic acid.

**Table S5.** Factors associated with HCV treatment among Aboriginal and Torres Strait Islander ETHOS Engage participants who had evidence of past or current HCV and who had injected in the previous month (N=178)

| **Characteristic** | | **Previous or current HCV infection,  n (col%)** | **History of HCV treatment,  n (row%)** | **OR (95% CI)** | **aOR (95% CI)** |
| --- | --- | --- | --- | --- | --- |
| **Total** | | 178 | 107 (60%) |  | |
| Age at enrolment | <45 | 101 (57%) | 53 (52%) | -ref- | -ref- |
|  | ≥45 | 77 (43%) | 54 (70%) | 2.12 (1.14, 3.97) | 2.26 (1.13, 4.50) |
| Gender | Man | 109 (61%) | 65 (60%) | -ref- | -ref- |
|  | Woman | 68 (38%) | 41 (60%) | 1.03 (0.55, 1.91) | 1.39 (0.68, 2.82) |
|  | Transgender/other^*^ | 1 (1%) | 1 (100%) | omitted | omitted |
| Location of clinic | Major cities of Australia | 138 (78%) | 82 (59%) | -ref- |  |
|  | Regional Australia | 40 (22%) | 25 (63%) | 1.14 (0.55, 2.35) |  |
| Homeless | No | 154 (87%) | 98 (64%) | -ref- | -ref- |
|  | Yes | 24 (13%) | 9 (38%) | 0.34 (0.14, 0.83) | 0.35 (0.13, 0.94) |
| Opioid agonist treatment status | Never | 21 (12%) | 11 (52%) | -ref- | -ref- |
|  | Past | 35 (20%) | 21 (60%) | 1.36 (0.46, 4.06) | 1.15 (0.35, 3.78) |
|  | Current | 122 (69%) | 75 (61%) | 1.45 (0.57, 3.68) | 1.22 (0.44, 3.40) |
| Incarceration history | Never | 30 (17%) | 17 (57%) | -ref- | -ref- |
|  | History only | 99 (57%) | 60 (61%) | 1.17 (0.51, 2.69) | 1.28 (0.52, 3.12) |
|  | Recent | 49 (28%) | 30 (61%) | 1.21 (0.48, 3.04) | 1.80 (0.63, 5.12) |
| Excessive alcohol consumption** | No | 97 (54%) | 61 (63%) | -ref- |  |
|  | Yes | 80 (45%) | 45 (56%) | 0.76 (0.41, 1.39) |  |
| Frequency of injecting | Within last month, <daily | 91 (51%) | 60 (66%) | -ref- | -ref- |
|  | Within last month, ≥daily | 87 (49%) | 47 (54%) | 0.61 (0.33, 1.11) | 0.71 (0.36, 1.42) |
| Main drug injected in last month | Heroin | 66 (37%) | 40 (61%) | -ref- | -ref- |
|  | Other opioids | 18 (10%) | 12 (67%) | 1.30 (0.43, 3.90) | 1.03 (0.32, 3.25) |
|  | Methamphetamine | 89 (50%) | 51 (57%) | 0.87 (0.46, 1.67) | 0.97 (0.45, 1.91) |
|  | Other | 5 (3%) | 4 (80%) | 2.60 (0.27, 24.57) | 2.13 (0.21, 21.91) |
| Recruitment wave | Wave 1 (2018-2019) | 102 (57%) | 57 (56%) | -ref- | -ref- |
|  | Wave 2 (2019-2021) | 76 (43%) | 50 (66%) | 1.52 (0.82, 2.81) | 1.72 (0.88, 3.36) |

*other refers to any individual who did not identify as a man, woman, or as transgender; **not reported transgender/other.

aOR, adjusted odds ratio; CI, confidence interval; HCV, hepatitis C virus; OR, odds ratio.


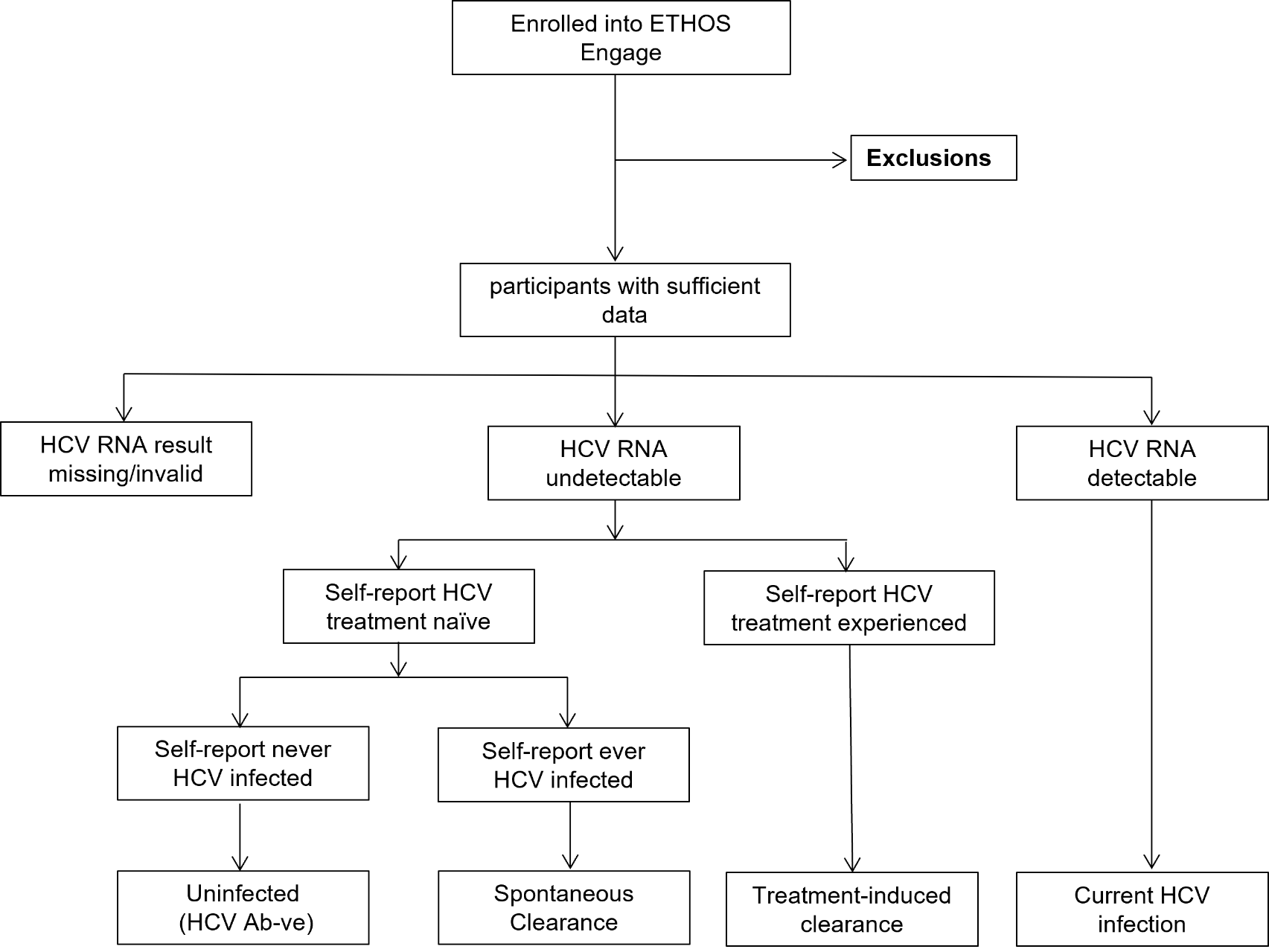


**Figure S1.** Derivation of current HCV infection. HCV, hepatitis C virus; RNA, ribonucleic acid.


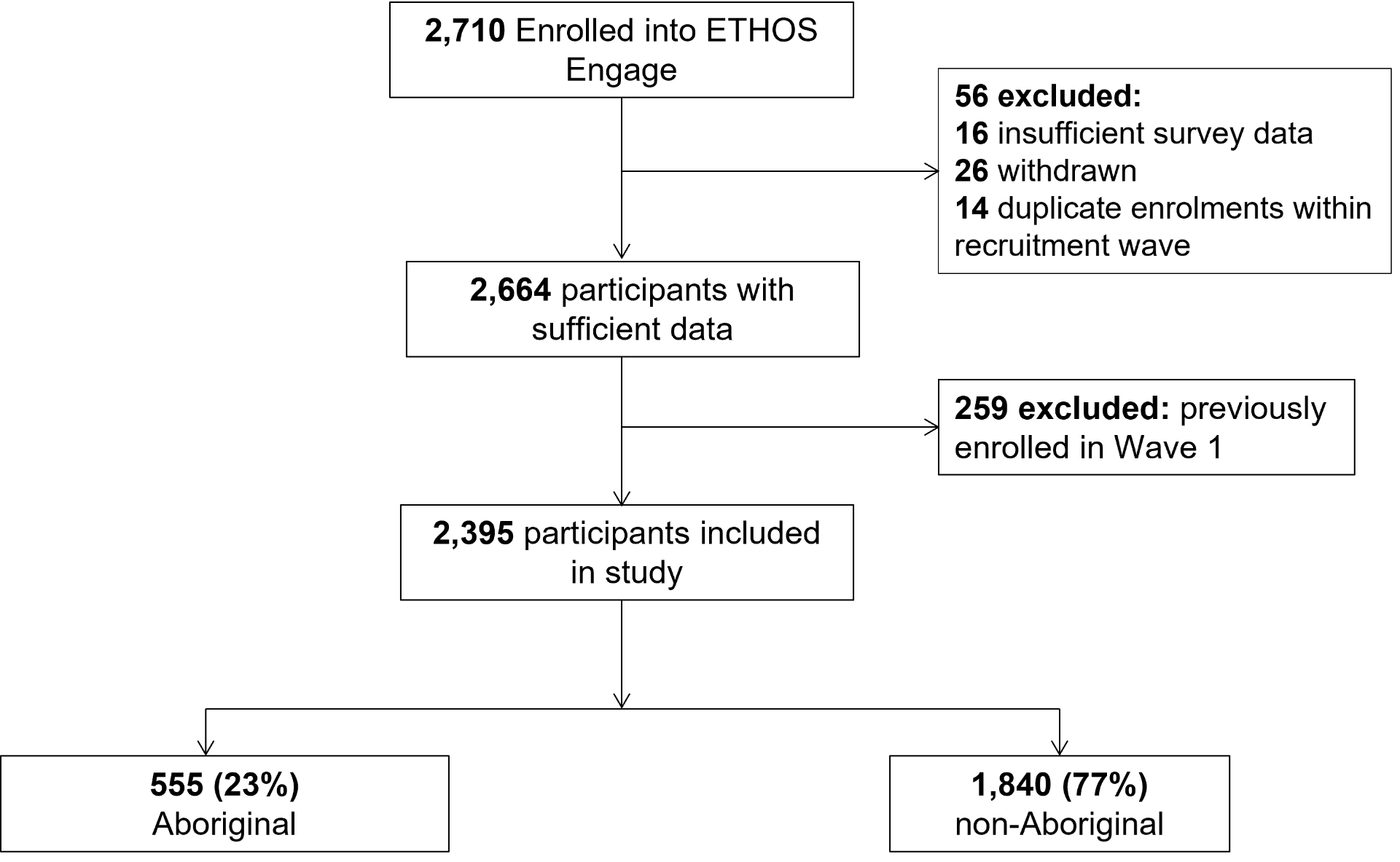


**Figure S2.** Cohort disposition, ETHOS Engage
